# Efficacy of brief dynamic interpersonal therapy in patients with major depressive disorder: a prospective, multicenter randomized controlled trial protocol

**DOI:** 10.1186/s13063-020-04569-8

**Published:** 2020-07-23

**Authors:** Lanlan Wang, Qian Wang, Wenhui Jiang, Jianfeng Luo, Jun Tong, Xiaosi Li, Fang Fang, Hongyan Wang, Wenqing Zhao, Diana Koszycki, Jianyin Qiu

**Affiliations:** 1 Shanghai Mental Health Center, Shanghai Jiaotong University Medical School, Shanghai, China; 2 Beijing Anding Hospital, Capital Medical University, Beijing, China; 3 Department of Biostatistics, School of Public Health, Fudan University, Shanghai, China; 4 Wuhan Mental Health Center, Wuhan, Hubei Province China; 5 Anhui Mental Health Center, Hefei, Anhui Province China; 6 Shanghai Hongkou District Mental Health Center, Shanghai, China; 7 University of Ottawa and Insitut du Savoir Montfort, Ottawa, Ontario Canada

**Keywords:** Major depressive disorder, Dynamic interpersonal psychotherapy, Multicenter randomized controlled trial

## Abstract

**Background:**

In China, psychodynamic psychotherapies are widely used as a treatment for depression. However, very few efficacy studies of psychodynamic therapies have been conducted with the Chinese population. This paper describes a study protocol of a multicenter randomized controlled trial of dynamic interpersonal psychotherapy (DIT), a brief manualized depression-focused intervention, in Chinese adults with major depressive disorder (MDD).

**Methods:**

Recruitment is planned in five hospitals. Two hundred forty patients with MDD will be randomly allocated on a 1:1:1 basis to either medication plus DIT, medication plus an active control psychotherapy, or medication alone. Patients will be assessed at baseline and at weeks 2, 4, 8, 12, and 16 during the acute treatment phase and 1, 3, 6, and 12 months posttreatment. The primary outcome is change from baseline in the 17-item Hamilton Depression Rating Scale, administered by independent raters who are blind to treatment allocation. The Hamilton Anxiety Rating Scale, Patient Health Questionnaire-9, Generalized Anxiety Disorder 7-item scale, response, remission and relapse rates, self-assessment of overall efficacy and satisfaction of patients, and side effect profiles are secondary measures.

**Discussion:**

This will be the first multicentered RCT in China to assess the efficacy of a brief psychodynamic intervention for MDD. The study has the potential to inform clinical treatment guidelines for the treatment of depression in China.

**Trial registration:**

ChiCTR, ChiCTR1800016970. Registered on July 5, 2018

## Administrative information

**Table:** 

**Title**	Efficacy of brief dynamic interpersonal therapy in patients with major depressive disorder: a prospective, multicenter randomized controlled trial protocol
**Trial registration**	ChiCTR, ChiCTR1800016970
**Protocol version**	The protocol version number is 2019-03 Date: 20191125
**Funding**	The study is funded by Shanghai Mental Health Center Clinical Research Center (Grant number CRC2017YB01)
**Author details**	1. Shanghai Mental Health Center, Shanghai Jiaotong University Medical School, Shanghai, China 2. Beijing Anding Hospital, Capital Medical University, Beijing, China 3. Department of Biostatistics, School of Public Health, Fudan University, Shanghai, China 4. Wuhan Mental Health Center, Hubei province, China 5. Anhui Mental Health Center, Anhui province, China 6. Shanghai Hongkou District Mental Health Center, Shanghai, China 7. University of Ottawa and Insitut du Savoir Montfort, Ottawa, Ontario, Canada
**Name and contact information for the trial sponsor**	Sponsor: Shanghai Mental Health Center Clinical Research Center Address: Wanping south road 600, Xuhui District, Shanghai, China Contact Name: Dr. Jun Chen Email: doctorcj2010@gmail.com
**Role of sponsor**	Review the research application, make decisions regarding funding, and oversee the study’s implementation. The sponsor will not have authority over data analysis, data interpretation, report writing. and submission of manuscripts for peer-review publication.

## Introduction

### Background and rationale

Major depressive disorder (MDD) is a prevalent psychiatric disorder with an estimated point prevalence of 10% worldwide and a lifetime prevalence up to 16% [1]. In China, the point prevalence of MDD ranges from 6.1 to 7.5% [2], with prevalence rates increasing annually. MDD tends to be a chronic and recurring illness and is associated with significant impairment in multiple domains and high suicide rates [3]. It is projected that by 2020, MDD will rank second among diseases with the highest social and economic burden worldwide [4]. The pervasiveness and disabling nature of MDD make the development of effective treatment protocols a high priority for the Chinese health care system.

In China, the treatment for MDD includes pharmacotherapy and psychotherapy, alone or in combination. Among the psychotherapies, psychodynamic therapy is widely used. Unlike other psychological interventions that resolve problems on a consciousness level, psychodynamic therapies focus on changing maladaptive personality structure and core schemas. While psychodynamic therapy is usually offered as a long-term intervention (i.e., 50 or more sessions) [5], this is costly and difficult to sustain within a public mental health care system. To increase accessibility and affordability of psychodynamic therapies, time-limited approaches have been developed in the last 30 years, with recent well-designed clinical trials demonstrating their efficacy in the treatment of depression [6–8].

Among the short-term approaches, dynamic interpersonal therapy (DIT) was recently developed as a specific depression-focused intervention that could be implemented within the context of a public health care system [9]. This 16-session manualized treatment is currently recommended as one of the psychotherapeutic approaches provided through the Improving Access to Psychological Therapies initiative in the UK [10, 11]. The development of DIT was influenced by object relations theory, theories of mentalization, attachment theory, and interpersonal theory, as well as clinical observation that the relationships of depressed patients are usually fraught with difficulties [11]. DIT conceptualizes depression as a response to perceived threats to attachments (loss/separation) and impaired mentalization function. The goals of treatment are to help patients understand the link between depressive symptoms and relational difficulties through identifying core, unconscious, recurring patterns of relating with others, to facilitate the capacity for self-reflection and understanding interpersonal behavior in terms of mental states, and to encourage new and adaptive ways of responding to challenges in interpersonal relationships [11]. The psychotherapist uses a range of established psychodynamic techniques, works with transferential reactions, and makes use of mentalization techniques. A description of DIT is provided in the “Methods” section of this paper.

Although little research has been conducted on the efficacy of DIT for depression, findings from available studies are promising. In an initial open pilot study of DIT, large pre- to posttreatment effect sizes were found for self-rated depression and anxiety. Further, 70% of patients had depression scores below clinical level at endpoint. This preliminary study also demonstrated that DIT was well accepted by patients, had face validity across a range of patients, and was easy to learn by experienced psychodynamically trained therapists working in routine clinical settings [12]. In another uncontrolled trial of DIT conducted in a primary care psychological service, 75% of patients reported a reduction in self-report measures of depression and anxiety. However, when using a reliable change index, which determines if the magnitude of change for a given participant is attributable to treatment and not due to chance, improvement was reduced to 42% for anxiety and 25% for depression [13]. In a more recent randomized controlled study, DIT was found to be comparable to CBT and superior to a control intervention in reducing depressive symptoms, with treatment gains maintained at the 12-month follow-up [14].

Since DIT and other psychodynamic therapies have been developed and evaluated in Western countries, findings cannot be readily generalized to China due to possible cultural effects. Thus, before promoting a new method of psychotherapy in Chinese culture, it is necessary to first test its applicability and make corresponding modifications if needed. The DIT manual developed by Lemma et al. was recently translated into Chinese [15]. The proposed study is the first in China to evaluate the efficacy of DIT for MDD in hospital settings, as well as one of the first multicentered RCT in China to assess the efficacy of a psychodynamically oriented psychotherapy for MDD. The results of this study will have important implications for the provision of evidence-based care for depression in China.

### Objectives

The objective of this trial is to determine whether medication plus DIT has greater efficacy than medication plus a control psychotherapy and than medication alone in the treatment of MDD. Medication treatment will consist of standard medication management for depression. The control psychotherapy, general supportive therapy (GSP), will be used to determine if DIT has benefits over and beyond common therapeutic factors that contribute to therapy outcome, such as therapist attention and support, expectation of improvement, and feeling understood. We hypothesize that DIT will be superior to the other treatment groups in improving symptoms of depression and anxiety at post-intervention and at follow-up.

### Trial design

This study is a multicenter, parallel, superiority, three-arm, randomized controlled trial with recruitment planned in five hospitals. Two hundred and forty outpatients will be randomly assigned on a 1:1:1 basis to one of the three treatment conditions, with 80 patients assigned to each group. Patients will be blind to the purpose of the study, and our primary efficacy measure will be administered by independent raters who are blind to treatment allocation.

## Methods: participants, interventions, and outcomes

### Setting and recruitment

Recruitment will take place in five hospitals in China. The sites include the Shanghai Mental Health Center, Beijing Anding Hospital, Anhui Mental Health Center, Wuhan Mental Health Center, and Shanghai Hongkou District Mental Health Center. New outpatients at each of the five sites will be pre-screened individually for 15–20 min by a research assistant (a senior psychiatric nurse working in the triage station). Those who are potentially eligible will be invited to a screen visit for confirmation of DSM-5 MDD based on the Mini International Neuropsychiatry Interview (MINI) [16] and other eligibility criteria. The MINI will be administered by qualified raters at each center (junior researchers with a background in psychiatry) who have been trained to high levels of inter-rater reliability (kappa = 0.85). Training in the MINI includes viewing teaching videos of an expert administering the MINI and as well as assessment of patients under the supervision of a qualified trainer.

**Fig. 1:**
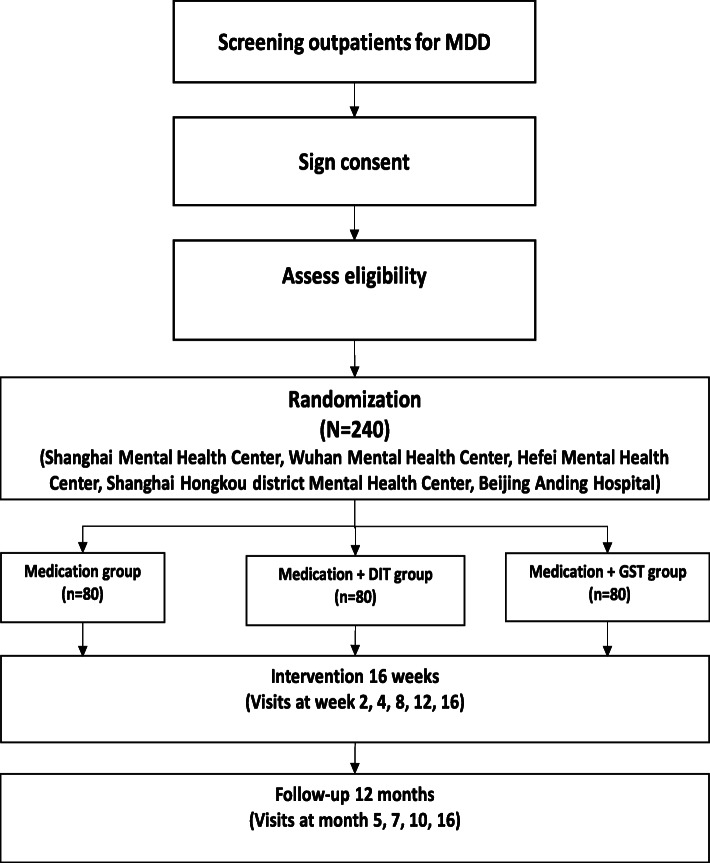
Flow diagram of enrollment, intervention, and assessments

Written informed consent will be obtained during the screen visit by trained research assistants. Patients will be informed that their participation is voluntary and that they can withdraw from the study at any time without any negative consequences to their care. All efforts will be made to protect the privacy of the patients. Possible adverse reactions will be monitored throughout the trial. The trial will be implemented based on the principles of good clinical practice and reported according to the CONSORT statement [17, 18]. The trial flow diagram is shown in Fig. 1. The Standard Protocol Items: Recommendations for Interventional Trials (SPIRIT) [19] checklist is shown in Additional file 1. This study has been registered at ChiCTR (ChiCTR1800016970).

### Participants and eligibility

To be eligible, patients need to be between the ages of 18–65 years, meet DSM-5 criteria for MDD with a score ≥ 18 on the 17-item Hamilton Depression Rating Scale (HAMD-17), have completed at least primary school education (i.e., ≥ 6 years of education), be antidepressant drug naïve or have discontinued antidepressant medication at least 8 weeks prior to the screen visit, have not received psychotherapy in the last 6 months, have not received other treatments for depression such as transcranial magnetic stimulation and electroconvulsive therapy, and provide written informed consent.

Exclusion criteria include a severe concurrent medical condition, significant visual and auditory deficits, lifetime history of psychosis or bipolar disorder, history of substance use disorders in the last 24 months, history of psychotic features of affective disorder, high suicide risk (MINI suicidality item score ≥ 10), severe personality disorders (e.g., cluster A and antisocial personality disorder), and intellectual disability. Other psychiatric comorbidities will be allowed so long as the MDD is the primary and predominant disorder.

### Randomization and blinding

Patients will be assigned to one of the three treatment groups via remote access to the central randomization procedure that is hosted by the Clinical Trial Center of the Shanghai Mental Health Center. Permuted blocks of randomly varying lengths will be used to ensure there is an equal number of patients in the 3 treatment groups at all times. Once a patient is deemed eligible, a research assistant at each site who is not involved in the recruitment process will log into a web-based interface to obtain the next allocation. This is restricted access, and users other than the system owner do not have access to future allocations. Sequence generation is therefore concealed from the study investigators, patients, and assessors.

Allocation of patients to each of three groups will be recorded in a separate assessment log used only by the research assistant. As blinding of patients, providers, and research assistants is not possible in psychotherapy trials, our primary outcome will be administered by independent evaluators who are unaware of the treatment patients are allocated to. The success of the blind will be ascertained by asking the evaluators to document whether patients revealed which group they were assigned to. In addition, patients will be asked not to reveal their assigned treatment during the blind assessments. The principal researchers and statisticians will also be blind to group allocation.

### Intervention description

#### Medication

Patients in all three treatment groups will be prescribed a selective serotonin reuptake inhibitor (SSRI) approved by the Chinese Food and Drug Administration (CFDA) for the treatment of MDD (e.g., paroxetine, sertraline, citalopram). The type of SSRI will be based on clinician recommendation, and the dose will be titrated according to usual practice. The dosage could be adjusted on a weekly basis over the 16 weeks of acute treatment if needed, with the maximum dose not exceeding dosage guidelines. Patients with sleep disorders will be prescribed benzazem if needed, but not for more than 2 weeks. Other psychotropic drugs and herbal remedies for depression are proscribed. Patients will be asked to keep a daily record of antidepressant medication use and dosage as well as any concomitant treatments. Medication use will be documented during the scheduled assessments.

#### Dynamic interpersonal psychotherapy

Patients assigned to the medication + DIT group will receive 16 weekly sessions of DIT, with each session lasting 45 min. A Chinese version of the DIT manual developed by Lemma et al. [15] will be used. The therapy has three phases with each phase associated with specific tasks. The principal therapeutic task of the initial phase of treatment (sessions 1 to 4) is to identify one dominant and recurring unconscious interpersonal pattern, called the “interpersonal affective focus” (IPAF), that is considered salient to the patient’s current depressive symptoms. The middle phase of therapy (sessions 5 to 12) is devoted to helping the patient work through the IPAF. During this phase, the therapist maintains a systematic focus on the agreed-upon IPAF by prioritizing the discussion of the patient’s current relationships that activate the IPAF, uses the therapeutic relationship as a live example of the IPAF in action, and helps the patient understand mental states as they relate to the week’s events and to the identified IPAF. The final sessions (sessions 13–16) focus on exploring feelings about ending therapy, reviewing treatment gains, and anticipating future difficulties and vulnerabilities.

#### General supportive psychotherapy

Patients assigned to the medication + GST group will receive 16 weekly sessions of GST, with each session lasting 45 min. As a control psychotherapy, supportive therapy is an active but less specific intervention that is intended to control for common factors that account for positive outcomes with all psychotherapies. GST is unstructured, and no specific psychotherapeutic techniques are used other than those common to all approaches (e.g., reflective listening, offering support, helping patients feel understood). GST is procedurally distinct from DIT, thus avoiding overlap between the study treatments. A manual for GST developed by our own research team will be followed in this trial and is available on request.

#### Therapist selection and supervision

The therapies will be delivered by licensed psychotherapists with at least 3 years of psychotherapy experience. Therapists will be master’s or doctoral-level clinical psychologists or psychiatrists. For DIT, we will select therapists who have at least 3 years of experience conducting psychodynamic psychotherapy. The therapists will deliver only one treatment to avoid treatment contamination, and they will be specifically trained in their respective treatment approach. Prior to the study, therapists will receive training from experts in DIT and GST. After training, only those who demonstrate competence in DIT and GST will serve as study therapists. To maintain treatment integrity throughout the trial, therapists at each site will receive 90 min of group supervision on a biweekly basis. Supervision will be provided by experts in the respective therapies.

#### Treatment integrity

All therapy sessions will be videotaped, and for each therapist-patient dyad, three tapes will be randomly selected from the early, middle, and late phase of therapy to examine therapist adherence and competence. Treatment adherence and competence will be assessed by two raters who are experts in the respective therapy. DIT adherence and competence will be evaluated with a Chinese version of a therapist competence scale developed by Lemma et al. [15]. GSP adherence and competence will be evaluated with a scale developed by the authors of this study.

#### Treatment discontinuation

Patients will be withdrawn from the study for the following reasons: (1) emergence of a new concurrent medical disease that is not well controlled, (2) exacerbation of a concurrent medical condition, (3) treatment-emergent serious adverse events, (4) the patient requests to be withdrawn, (5) poor treatment compliance, and (6) the researchers believe it is in the best interest of the patient to discontinue treatment (e.g., increased suicide risk, participation in the study presents a significant burden to the patient, patient requires hospitalization or day treatment). The decision to withdraw a patient from the study will be made by the site principal investigator in consultation with the research team at the lead research site.

### Outcomes measures

#### Primary efficacy measure

Our primary efficacy measure is the HAMD-17 administered by independent raters who are blind to treatment allocation.

#### Secondary efficacy measures

Rates of response, remission, and relapse will be based on the HAMD-17. Response is defined by a 50% reduction (or more) in total HAMD score, and remission is defined as a HAMD score ≤ 7. Relapse is defined as an episode of at least 1-month duration meeting DSM-5 criteria for MDD. Other secondary measures include the Hamilton Anxiety Scale (HAMA-14), which will be administered by blind evaluators; the Patient Health Questionnaire (PHQ-9); Generalized Anxiety Disorder 7-item scale (GAD-7); and self-assessment of overall efficacy and satisfaction of patients. These measures will be administered at the same time points as the HAMD-17.

#### Participant timeline

**Fig. 2:**
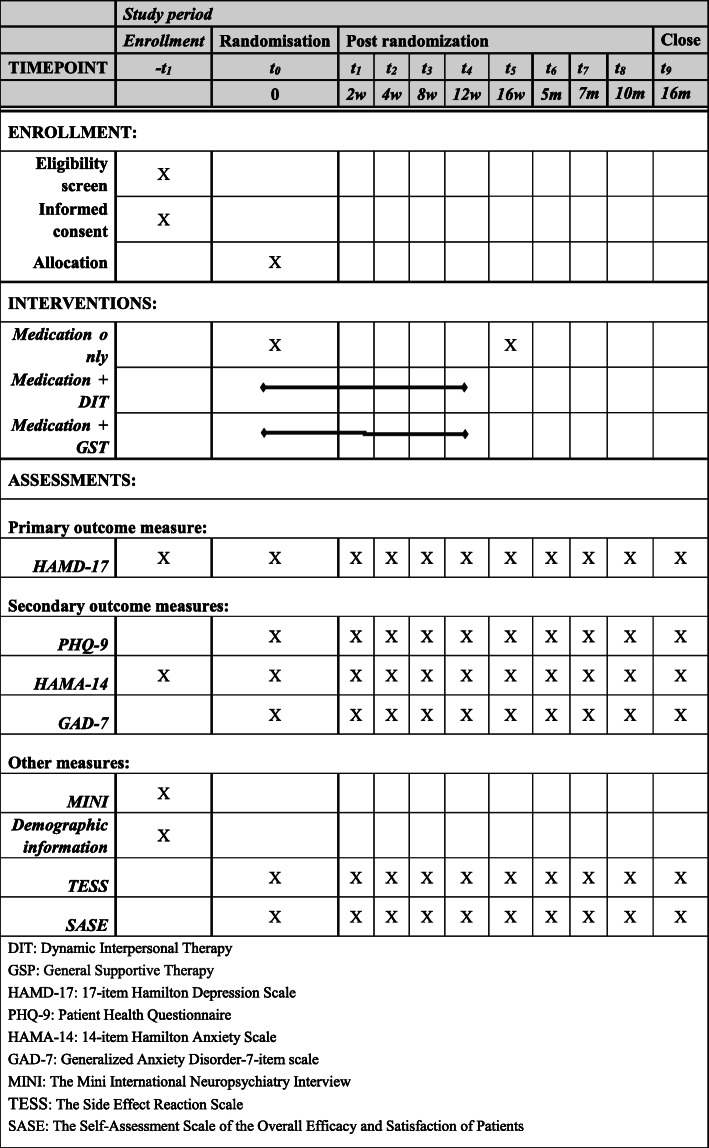
Study design and measurement time points

The intervention cycle will be 16 weeks. Acute treatment efficacy will be assessed at baseline and at weeks 2, 4, 8, 12, and 16. Follow-up assessments will be conducted at 1, 3, 6, and 12 months posttreatment. An overview of specific measures and time points for data collection is presented in Fig. 2.

### Sample size

A power analysis was conducted by the program “G*Power.” Twenty-six studies of the effectiveness of short-term dynamic therapy (STDT) were subjected to two meta-analyses. Effect sizes (ES) obtained by each method were similar. STDT attained average ESs of 0.71 and 0.34, relative to waitlist and minimal treatment groups, respectively, with a slight superiority at long-term follow-up assessment [20]. Based on these former theoretical considerations and results of comparable studies [21, 22], we assume a medium effect size of 0.50. An a priori power analysis with 80% power using a two-tailed test at the 5% level of significance indicated that 64 subjects per treatment group will be adequate to detect this difference. Assuming a dropout rate of about 20%, the target enrollment is 240 patients.

### Data collection and management

The project team at the Clinical Psychological Department of the Shanghai Mental Health Center will serve as the data coordinating center responsible for data collection forms, coordination of data transfer, and data analysis. Patients will be under close medical supervision throughout the trial. If any adverse events emerge, and in the unlikely event that harm is suffered, the research management team will liaise with the clinician responsible for the care of the patient. All adverse events will be documented in the final written report of this study. All study data will be stored securely at the Shanghai Mental Health Center. Paper-based documents and data will be stored in a secure filing cabinet, and electronic data will be secured on a password-protected computer. All documents that contain names or personal identifying information will be stored separately from other study data and identified by code number. Access to files will be limited to research staff involved in the study. The statistician performing the data analyses will receive depersonalized data where the participants’ identifying information will be replaced by an unrelated sequence of numbers.

### Statistical analysis

The principal analyses will be conducted using the intention-to-treat sample. Two-sided 5% significance level will be adopted, and corresponding 95% CIs will be calculated whenever possible. Primary and secondary continuous outcomes from baseline to week 16 will be analyzed using analysis of variance and general linear mixed models. Treatment group, time, and interaction between treatment and time will be the main predictors of interest, with gender, age, education level, and baseline scores entered as covariates and treatment site as a random effect in the model. The mixed models will be estimated using restricted maximum likelihood. For the main analysis, the least square mean difference between the groups at 16 weeks will be tested; additional analyses will include comparisons at weeks 2, 4, 8, and 12, with adjustment for multiple testing. Multiple comparison issues of post hoc analysis will be handled using Bonferroni adjustment. Dichotomous outcomes will be compared between groups using chi-square test, Fisher’s exact test, or generalized estimating equation. To assess maintenance of treatment gains, the above analyses will be repeated with the addition of follow-up data (i.e., 1, 3, 6, and 12 months posttreatment).

### Trial oversight

The day-to-day management of the trial will be the responsibility of the project management team, based at the Shanghai Mental Health Center. The team will meet monthly to assess progress of the study. The project manager will also be responsible for training the research assistants at each of the trial centers. The trial statistician will be closely involved in setting up the data capture systems and databases and in designing the clinical reporting forms.

A Trial Steering Committee (TSC) and Data and Safety Monitoring Committee (DSMC) will be established to guarantee the quality of study. The TSC will be responsible for overseeing the conduct of the study. It will have regular conference calls and face-to-face meetings at start-up, 50% recruitment, and when data collection is completed. Topics for discussion might include patient enrollment, progress on data collection, patient retention, protocol amendments, protocol violations, adverse events, and timely reporting of trial results. Impromptu meetings will be called to deal with unforeseen difficulties. The TSC will also provide advice, through its chair, to the principal investigator and trial sponsor on all appropriate aspects of the trial and make decisions as to the future continuation of the trial.

Treatment-emergent adverse events will be monitored at each visit. Adverse events are defined as any significant unfavorable change in the patient’s pretreatment mental condition regardless of its relationship to treatment. Serious adverse events include mortality, hospitalization, suicide, or attempted suicide. All adverse events will be recorded in the case report form. The assessment of adverse events includes classification, grading, identifying the relationship with treatment, and formulating coping methods.

The trial will also be monitored or audited in accordance with the current approved protocol, relevant regulations, and standard operating procedures by the study sponsor. A monitoring plan will be developed according to standard operating procedures, which involve a risk assessment. The monitoring activities are based on the outcome of the risk assessment and may involve central monitoring and site monitoring.

### Dissemination plan

Patients who are interested in the trial results will be informed of the findings. Results will be published in high-impact scholarly journals and disseminated through national and international scientific meetings. No professional writers will be employed for publications associated with this study. The authors are intended to participate in future publications associated with this protocol.

## Discussion

DIT is a brief psychodynamic psychotherapy that shows promise for the treatment of depression. The current study will be the first multicentered RCT of DIT conducted in China. The study will provide valuable information about the relative efficacy of DIT compared to medication plus an active control psychotherapy and medication alone and has the potential to inform clinical treatment guidelines for this disorder in China.

## Trial status

The trial is in the ongoing recruitment phase. The protocol version number is 2019-03 (20191125). Recruitment began on April 8, 2019. It is estimated that recruitment will be completed by April 30, 2021.

## Supplementary information

**Additional file 1.** SPIRIT 2013 Checklist.

## Data Availability

The datasets used and/or analyzed will be available upon request to the corresponding author. All authors will have access to the complete dataset.

## Abbreviations

DIT: Dynamic interpersonal therapy
MDD: Major depressive disorder
HAMD-17: 17-item Hamilton Depression Scale
HAMA-14: 14-item Hamilton Anxiety Scale
PHQ-9: Patient Health Questionnaire
GAD-7: Generalized Anxiety Disorder 7-item scale
TESS: The Side Effect Reaction Scale
SASE: The Self-Assessment Scale of the Overall Efficacy and Satisfaction of Patients
RCT: Randomized controlled trial
GSP: General supportive therapy
MINI: The Mini International Neuropsychiatry Interview
SSRI: Selective serotonin reuptake inhibitor
CFDA: Chinese Food and Drug Administration
IPAF: The interpersonal affective focus
STPP: Short-term psychodynamic psychotherapy
TSC: Trial Steering Committee
DSMC: Data and Safety Monitoring Committee
SAEs: Serious adverse events
ICMJE: International Committee of Medical Journal Editors
